# Identifying future models for delivering genetic services: a nominal group study in primary care

**DOI:** 10.1186/1471-2296-6-14

**Published:** 2005-04-14

**Authors:** Glyn Elwyn, Adrian Edwards, Rachel Iredale, Peter Davies, Jonathon Gray

**Affiliations:** 1 Centre for Health Sciences Research, Cardiff University, 56 Park Place, Cardiff, CF10 3AT, Wales; 2 Institute of Medical Genetics, Cardiff University, Heath Park, Cardiff, CF14 4XN, Wales; 3 Primary Care Group, University of Wales Swansea, SA2 8PP, Wales

## Abstract

**Background:**

To enable primary care medical practitioners to generate a range of possible service delivery models for genetic counselling services and critically assess their suitability.

**Methods:**

Modified nominal group technique using in primary care professional development workshops.

**Results:**

37 general practitioners in Wales, United Kingdom too part in the nominal group process. The practitioners who attended did not believe current systems were sufficient to meet anticipated demand for genetic services. A wide range of different service models was proposed, although no single option emerged as a clear preference. No argument was put forward for genetic assessment and counselling being central to family practice, neither was there a voice for the view that the family doctor should become skilled at advising patients about predictive genetic testing and be able to counsel patients about the wider implications of genetic testing for patients and their family members, even for areas such as common cancers. Nevertheless, all the preferred models put a high priority on providing the service in the community, and often co-located in primary care, by clinicians who had developed expertise.

**Conclusion:**

There is a need for a wider debate about how healthcare systems address individual concerns about genetic concerns and risk, especially given the increasing commercial marketing of genetic tests.

## Background

'Imagine what it would be like if doctors could look at your medical future' says an advert in the Harvard Business Review [1]. If companies are already marketing the possibility of a genetic manipulated future, is it not time doctors considered how to manage what will become a growing area of work? Genetics will alter the face of medicine, as the inherited components of common diseases and cancers are uncovered and patients' awareness of susceptibility increases. This trend is likely to lead to more individuals approaching primary care for guidance, for genetic risk assessment or counselling and perhaps testing [2,3]. Many practitioners are confident that generalists can absorb this demand by increasing their knowledge and making use of new software [4,5]. Others are sceptical and fear primary care clinicians will not want the additional task of providing genetic advice [6], adamant that this new role – both in time and effort – goes beyond what can be realistically provided. Patients are not concerned with such debates: they look for convenient access to effective services [7]. Should primary care respond to these demands and if so, how? If not, are we guilty of allowing technological developments in predictive testing and commercial pressures overtake the ability of services to react appropriately, leaving individuals with genetic concerns unsupported? [8,9]

Genetics clinics are experiencing workload increases [10]. Referrals to cancer genetic services are a particular area of demand as predictive genetic testing for breast, ovarian and colorectal cancer becomes more widely known [10]. To meet this demand, genetic services are experimenting with new methods of service delivery. A number of models are reported and include initiatives such as initial risk assessments by mailed questionnaire or telephone interview or the use of letters to transmit risk information to referred patients without brining them to clinic [11,12]. Some services are examining the use of non-medical genetic counsellors to assess family pedigrees and are using nurses to undertake specialised genetic counselling. Some services have explored the use of video-conferencing in order to increase access to populations that live in rural areas [13]. Nevertheless, the basic service delivery model is still one predicated on specialist care: general practitioners refer patients to the secondary (or tertiary) care sector. As yet, there has been no guideline that indicates that some genetic issues should be managed in primary are, although guidelines are emerging that ask generalists to stratify risk by categorising patients according to risk and to only refer those above a suggested threshold. However, as demand for genetic services continues to grow, the viability of this service delivery model needs to be examined.

As elsewhere in the UK, genetic services in Wales are provided at a regional level, using a distributed clinic approach in three centres (North, West and the South East). Referrals are accepted from a range of sources, and for cancer genetic referrals a triage system involving postal questionnaire and telephone assessment is used to stratify patients into low, medium and high-risk groups. Given increasing demand however, there is speculation that genetics could be integrated into community settings, perhaps in family practice, close to kinship relationships and in a context of continuous care. However, there are also concerns about patients living 'at risk' with no obvious means of support [10]. Genetic risk assessment is a task requiring a detailed history, and where there are uncertainties regarding neoplasm in other family members, validation of disease by pathological confirmation is necessary. To determine individual risk, family members have to be contacted and blood samples obtained for DNA analysis from living affected relatives. This process is lengthy, complex and depends on effective communication between health care professionals, their patients, family members and between health professionals themselves. Is it feasible that primary care services can be re-designed to enable genetic risk assessment and counselling for those identified as moderate to high risk to take place and then referred onwards to the appropriate specialist service?

This study aimed to enable general practitioners to generate possible delivery models for genetic services and then critically assess these different possibilities over the next five to ten years. To facilitate the emergence of a wide range of options and possibilities a modified nominal group technique was employed.

## Methods

General practitioners were invited to discuss developments in genetics, emphasising cancer genetics, and to consider how services could be re-designed to meet anticipated growth in patient demand for genetic advice, counselling and further management. To obtain a varied sample, meetings were arranged in three different locations (Swansea, Newport and Cardiff). Two mailings were circulated to all practitioners in the relevant catchment areas using details available to the postgraduate offices. Approximately 150 practitioners were mailed each area.

The meetings were described as having two broad aims: firstly, to inform general practitioners about developments in genetics and how these might translate into patient concerns. Secondly, to involve practitioners in considering how genetic services could best meet this anticipated increased demand. The meetings took place between March and September 2003, at broadly the same time as the implementation of the new contract for general practice in the UK [14].

The structure of the meetings was standardised. A specialist in medical genetics (JG) provided an overview of recent advances in genetics and advised participants of the developments in predictive testing and pharmacogenetics. The predicted increasing demand for genetic advice was discussed. The presentation described the process of assessing family history, verifying verbal reports of diseases in other family members, assessing and communicating genetic risk to patients.

After this presentation, a modified nominal group technique was used [15]. Participants were asked to write, without conferring, a list of possible service delivery models that could be designed to provide genetic services to patients. Participants were asked to think creatively about news kinds of services and, for the purposes of the exercise, to put aside concerns about funding or the future of general practice. A facilitator then asked each participant to describe the models they proposed, one at a time, and they were outlined on flip charts. At this stage, the facilitator (GE) clarified and categorised the models, to remove duplications and arrived at an agreed list of service delivery designs. After this list had been agreed, participants were asked to conduct brief (10–20 minute) small group discussions in order to discuss the advantages and disadvantages of each model. Each practitioner then independently ranked the agreed models and the results were shared and discussed. In order to compare the results of the meetings, rankings given to different service delivery models were given inverse scores, and totals calculated to determine overall rankings.

## Results

Three events were held at which a nominal group process was undertaken immediately after a short standardised presentation about recent developments in genetics and cancer genetics. A total of 37 practitioners participated: they represent practitioners who were mostly in the mid-careers and were from a range of practice sizes. Compared to other similarly advertised events, attendance was approximately 50% lower: details of the attendees are provided in Table 1.

**Table 1:** Details of general practitioner participants at three nominal group events

**Meeting Area**	**Number at meeting**	**Gender, (Mean number of years in practice)**	**Mean Practice Size (Whole Time Equivalent Doctors)**
South West Wales	13	9 male, 4 female (17).	5
South East Wales	9	5 male, 4 female (13).	2
Cardiff	15	6 Male, 6, female, 3 not specified, (14).	4.6

Participants proposed lists of service models ranging from patient self-referral to telephone-based services such as NHS Direct, to maintaining existing arrangements where general practitioners continue to act as gatekeepers to other services. After clarifying the nature of each proposed model, the list was summarised and distinctive service delivery models were given a short descriptive names. Small group discussions were conducted to examine their pros and cons. At this point, each individual practitioner was asked to rank his or her preferred service models. The hypothetical service delivery models, their ranking at each meeting and overall ranking are provided in Table 2.

**Table 2:** Genetic service delivery models proposed and ranked

**Service Models**	**Meeting 1**		**Meeting 2**		**Meeting 3**		
	
	*Rank*	*Score*	*Rank*	*Score*	*Rank*	*Score*	**Score Total**
**Community based service **provided by genetic counsellors, not managed by general practice, but could be located in practices or local community centres to provide local patient assessment and advice.	1	8	1	8	3	6	**22**
**Enhanced primary care**: a service located within primary care, with specialists in genetic risk assessment, with support made possible by information technology and software applications.	2	7	3	6	1	8	**21**
**Special 'genetic' clinics**: this model was suggested so that the privacy and discretion analogous to 'genitourinary clinics' was built in, and where self-referral is possible and anonymity and confidentiality respected.	3	6	4	5	2	7	**18**
**Traditional gatekeeper model: **where general practitioners undertake an initial assessment, using standardised referral guidelines, and refer patients who are not categorised as 'low' risk.	4	5	2	7	4	5	**17**
**Direct access telephone service**: a 'genetics direct' model where patients have their genetic pedigrees assessed by counsellors with assess to pedigree software tools.	5	4	5	4	5	4	**12**
**Drop in service **for genetic assessment: e.g. similar to the Citizen Advice Bureau model.	6	3	-	-	-	-	**3**
**Private service**: patients with concerns are directed to commercial providers either in the UK or elsewhere.	7	2	-	-	-	-	**2**
**Pharmacy led service**: patients with concerns are directed to pharmacists, who could also undertake pharmacogenetic profile testing and offer lifestyle advice.	8	1	-	-	-	-	**1**

Table 2 illustrates the range of possible models generated during the nominal groups and shows that general practitioners are willing to consider a range of service delivery methods. Compared to the other two meetings, practitioners at the first meeting (south west Wales) generated many innovative approaches, including a telephone-based service integrated with an on-line family pedigree software and a suggestion that community pharmacists might wish to develop a role as genetic counsellors. Another suggestion was a 'drop in' centre as an attempt to 'de-medicalise' the assessment of an individual's genetic make-up. Some practitioners were aware of commercial companies selling genetic tests on the Internet and proposed that any initiatives linked to these should be privately funded and not covered as part of the state funded health care system.

The results of the ranking exercise showed all participants were agreed on one point: that the current gatekeeper model of primary care was not going to be able to deliver the genetic assessment, counselling and possibly testing that patients would require. However, participants felt genetic services should remain close to primary care, either in community settings or co-located and delivered by practitioners with specialist skills in this area, such as a general medical or nurse practitioner with a special interest. These models, including the current delivery model, were ranked higher than innovations based on telephone-based assessment alone or those located in different contractor professions. No model was a clear favourite among all three groups.

## Discussion

### Principal findings

Those general practitioners who accepted the invitation to hear about genetic developments iand take part in the nominal group process did not believe current systems were sufficient to meet anticipated patient demand for genetic services. Within the group process practitioners proposed a wide range of different service options, although no single option emerged as a clear preference for those participating. Surprisingly no argument was put forward for genetic assessment and counselling being central to family practice, neither was there a voice for the view that the family doctor should become skilled at advising patients about predictive genetic testing and be able to counsel patients about the wider implications of genetic testing for patients and their family members.

The views emerging from the groups reflected those of practitioners who are in routine NHS practice and therefore may not be considering the wider or future impact of *not *undertaking genetic assessment in primary care. Nevertheless, all the preferred models put a high priority on providing the service in the community, and often co-located with general practice. Although not directly addressed in the suggested service models, it was clear that the general practitioners had an open mind about which professional group would be best placed to undertake genetic counselling. Their understanding of the current secondary care model was that nurses with special training undertake the assessments.

### Strengths and weaknesses

The use of the nominal group process is strength of this study. It is a recognised means of allowing participants to give free rein to ideas, without constraint. The only limitation on the process is the knowledge and experience of those taking part in the group. It was unfortunate that the sample size was small. On the basis of prior attendance at postgraduate events similar to this one, 60 participants had been expected. The small sample size may be explained by the subject being given a low priority: this area of practice is not yet seen as having urgency in the mind of service based general practitioners. Participants had noted a slight increase demand for genetic advice, particularly among women concerned about breast or ovarian cancer, however increased requests for other types of predictive genetic testing had been experienced. Some will regard the non-specialist perspective of this work as a weakness but the study was purposefully designed to obtain the preferences of service-based general practitioners as a 'bottom-up' exercise to identify the delivery models felt to be appropriate and applicable in the evolving primary care context.

### Results in context

Placed in the context of publications that describe the expected impact of predictive genetic testing [16,17] there are few studies in which the effectiveness of different service delivery models has been examined. Holloway reported a study demonstrating the economy of using postal questionnaires compared to specialist nurse interviews as a means of assessing familial breast cancer risk [12]. Campbell reported a cluster randomised trial of a GP based genetic clinic, versus the normal practice of referral to a regional service and showed a larger increase in referral rates when clinics were based in primary care and that patients from the GP clinic had an inappropriate level interest and expectation of the appropriateness of genetic testing [10]. Elwyn reported reactions to a nurse-led triage system [18]. These studies did not consider other possible service delivery models. The work reported in this study provides a wider canvas of possible models and novel approaches. Genetic counselling and testing services need to combine accurate, comprehensive genetic risk assessment with methods that can be both local and sensitive to family contexts. It may be possible for general practice in the UK to develop expertise in genetic assessment, using the possibility of contracting for enhanced services.

## Conclusion

As a result of developments in genetics, there are increasing demands being made on primary care and genetic services to address patient concerns and manage requests for genetic advice, risk assessment and testing. Innovations in the ways services could be delivered have started to appear but there is a lack of discussion and planning about how the NHS intends to deal with the impact of the new genetics. General practitioners agree that the current referral processes and structures are unlikely to meet anticipated needs, but they do not have agreement about how services could be re-designed to meet anticipated demand. The practitioners suggested a range of potential service delivery models: the common thread among those which were ranked highest was that the service should be located close to communicates and work in close liaison, or embedded in, primary care provision. There is a need for a wider debate about how healthcare systems address how individual concerns about genetic risk are counselled and managed, especially given the likely commercial marketing of genetic tests. This study demonstrates that there is no obvious preferred solution to the problem of designing a service or system to provide genetic advice and assessment to an increasing number of patients and an implicit sense of scepticism about the likely impact of the new genetics, echoing other commentators [19]. It was also unclear how clinicians were expected to integrate genetic information about individuals and their relatives across an integrated electronic patient record shared between healthcare organisations – an area where concerns about data security and confidentiality abound. General practitioners are willing to suggest a range of models but there is no clear preference. From the groups, it was presumed that as a routine service, primary care practitioners would not undertake detailed genetic assessments but were open to the concept of a specialist in this area operating in a primary care arena. The practitioners did not differentiate between genetic concerns that were likely to be more frequent and therefore might be considered to become part of the primary care service, such as the stratification of women with a family history of breast or ovarian cancer into risk categories and then referring those who were calculated to be above population risk. Whether the aggregated views of this selected sample would find resonance among other stakeholders needs to be explored.

## Authors' contributions

GE had the original idea for the study and all authors developed the proposal. PD and GE led the nominal group sessions. GE, AE and PD contributed to the analysis and interpretation of the data. GE wrote the first draft of the paper and all authors contributed to subsequent drafts. GE acts as guarantor.

## Competing interests

The author(s) declare that they have no competing interests.

## Pre-publication history

The pre-publication history for this paper can be accessed here:



## References

[B1] (2004). Harvard Business Review.

[B2] Robins R, Metcalfe S (2004). Integrating genetics as practices of primary care. Soc Sci Med.

[B3] Rich EC, Burke W, Heaton CJ, Haga S, Pinsky L, Short MP, Acheson L (2004). Reconsidering the family history in primary care. J Gen Intern Med.

[B4] Watson EK, Shickle D, Qureshi N, Emery J, Austoker J (1999). The 'new genetics' and primary care: GPs' views on their role and their educational needs. Family Practice.

[B5] D G, Emery J, Walton R, Murphy M, Austoker J, Yudkin P, Chapman C, Coulson A, Fox J (2000). Computer support for interpreting family histories of breast and ovarian cancer in primary care: comparative study with simulated cases. BMJ.

[B6] Kumar S (1999). Resisting revolution: generalism and the new genetics. Lancet.

[B7] Iredale R, Brain K, Edwards L, Gray J, France E (2003). The information and support needs of women at high risk of familial breast and ovarian cancer: how can cancer genetic services give patients what they want?. Familial Cancer.

[B8] Kinmonth AL, Reinhard J, Bobrow M, Pauker S (1998). The new genetics: Implications for clinical services in Britain and the United States. BMJ.

[B9] Emery J, Hayflick S (2001). The challenge of integrating genetic medicine into primary care. BMJ.

[B10] Campbell H, Holloway S, Cetnarskyj R, Anderson E, Rush R, Fry A, Gorman D, Steel M, Porteous M (2003). Referrals of women with a family history of breast cancer from primary care to cancer genetics services in South East Scotland. Br J Cancer.

[B11] Phelps C, Platt K, France L, Gray J, Iredale R (2004). Delivering information about cancer genetics via letter to patients at low and moderate risk of familial cancer: a pilot study in Wales. Familial Cancer.

[B12] Holloway S, Porteous M, Cetnarskyj R, Anderson E, Rush R, Fry A, Gorman D, Steel M, Campbell H (2004). Patient satisfaction with two different models of cancer genetic services in south-east Scotland. Br J Cancer.

[B13] Gray J, Brain K, Iredale R, Alderman J, France E, Hughes H (2000). A pilot study of telegenetics. J Telemed Telecare.

[B14] General Practitioners Committee (2002). Your contract, your future.

[B15] Mays N and Pope C, Jones J, Hunter D (1999). Using the Delphi and nominal group technique in health services research. Qualitative Research in Health Care.

[B16] Harper PS, Hughes HB, Raeburn JA (1996). Clinical genetics services into the 21st century. Summary of a report of the Clinical Genetics Committee of the Royal College of Physicians. J R Coll Physicians Lond.

[B17] Knottnerus JA (2004). Community genetics and community medicine. Family Practice.

[B18] Elwyn G, Iredale R, Gray J (2002). Reactions of general practitioners to a triage controlled referral system for cancer genetics. Family Practice.

[B19] Holtzman NA, Marteau TM (2000). Will genetics revolutionize medicine?. N Engl J Med.

